# Fasting Blood Glucose-Based Novel Predictors in Detecting Metastases and Predicting Prognosis for Patients with PNENs

**DOI:** 10.3390/jpm14070760

**Published:** 2024-07-17

**Authors:** Li Yu, Mengfei Fu, Liu Yang, Hui Sun

**Affiliations:** 1 Department of Emergency Medicine, Union Hospital, Tongji Medical College, Huazhong University of Science and Technology, Wuhan 430022, China; m201775594@alumni.hust.edu.cn; 2 Department of Endocrinology, Union Hospital, Tongji Medical College, Huazhong University of Science and Technology, Wuhan 430022, China; d202181840@hust.edu.cn (M.F.); d202181880@hust.edu.cn (L.Y.)

**Keywords:** pancreatic neuroendocrine neoplasm, metastasis, fasting blood glucose-to-albumin ratio, fasting blood glucose-to-lymphocytes ratio, fasting blood glucose-to-hemoglobin ratio

## Abstract

Objective: To explore three novel fasting blood glucose (FBG)-based novel indicators, including the FBG-to-albumin ratio (FAR), FBG-to-lymphocytes ratio (FLR), and FBG-to-hemoglobin ratio (FHR), in predicting prognosis and detecting metastasis for patients with pancreatic neuroendocrine neoplasms (pNENs) after resection. Materials and Methods: A total of 178 pNENs patients who underwent surgical resection were included in this study. Receiver operating characteristic (ROC) curves were used to evaluate the diagnosis values of FAR, FLR, and FHR, and the cutoff values were obtained for further analyses. Univariate and multivariate analyses were conducted to determine the independent predictors. The Kaplan–Meier method was used to evaluate the progression-free survival (PFS) and overall survival (OS) of the pNENs patients. Results: The optimal cutoff values of FAR, FLR, and FHR were 0.17, 2.85, and 0.028, respectively. As for PFS, the area under the curve (AUC) was 0.693 for FAR, 0.690 for FLR, and 0.661 for FHR, respectively. The AUC was 0.770, 0.692, and 0.715 accordingly for OS. The groups with lower FAR, FLR, and FHR were significantly associated with prolonged PFS and OS (*p* < 0.05). In patients with metastasis, the lower FAR group was correlated with significantly longer PFS and OS (*p* = 0.022 and 0.002, respectively). The FLR was an independent predictor of PFS in pNENs patients, and the FAR was a predictor of OS. FAR was an independent indicator of PFS in patients with metastasis. Conclusions: Preoperative FAR, FLR, and FHR are effective in predicting the prognosis of pNEN patients and detecting the synchronous metastases.

## 1. Introduction

Pancreatic neuroendocrine neoplasms (pNENs) rank as the third most common neuroendocrine tumor subtype in the gastro-entero-pancreatic system [1,2]. The incidence presents a steady rise over the last few decades worldwide, with a five-year survival of 37.6% [3]. However, even in the pathologically well-differentiated tumors, the high heterogeneity of pNENs exhibits distinct biological behavior that affects the treatment decision and patient prognosis.

The classifications of pNENs according to the histological grade and the staging by American Joint Committee on Cancer (AJCC) have been widely applied in clinical practice [4]. These indices offer guidance on the course of treatment as well as insights into the patient prognosis. However, the AJCC staging system is based on the imaging examination, which is costly and often diagnosed pNENs occasionally when adopted for other diseases. Additionally, the prognostic value of this staging system is limited for patients without metastasis.

Laboratory tests are generally inexpensive and easily acquirable in clinic. Nonetheless, the prognostic value of hematological indices is frequently underestimated, particularly for those that appear to have no direct correlation with tumor behaviors. For instance, blood serum levels of alkaline phosphatase (ALP) and albumin (Alb) are not tumor biomarkers, but their ratio (ALP to Alb ratio, APAR) can be useful in predicting a patients’ prognosis for a variety of cancer types [5,6,7]. However, a previous study indicated that the APAR is insufficient to predict the overall survival (OS) of pNENs [5]. Therefore, it is imperative to explore more reliable indicators for these patients.

Recent studies have suggested that hyperglycemia may contribute to the cancer progression, though no substantial evidence indicates the possible link among them [8]. The prediction value of fasting blood glucose (FBG) for cancer patients is still under investigation due to the rarely relevant study. Additionally, the nutrition and inflammatory status of patients are linked to their outcomes. Some studies reported that the high C-reactive protein (CRP), low albumin, and low hemoglobin showed an increased risk trend of negative prognosis, but there are no significant differences [9]. These findings indicated that a single indicator is insufficient to predict the patients’ outcome.

Given these limitations, we conducted the present study to explore the value of three novel indicators, which are the FBG-to-albumin ratio (FAR), FBG-to-lymphocytes ratio (FLR), and FBG-to-hemoglobin ratio (FHR), for predicting the prognosis of pNEN patients and detecting their synchronous metastasis. As far as we know, there are no reports about these three indicators.

## 2. Materials and Methods

### 2.1. Patient Selection

Consecutive patients who underwent surgical resection and were pathologically diagnosed with pNEN at our center from 1 May 2010 to 31 December 2021 were reviewed. The exclusion criteria were as follows: patients who (a) are previously diagnosed with other malignant tumors; (b) complicated with serious comorbidities, such as severe dysfunction of the liver, kidney or heart; and (c) have missing data.

The demographic, clinical, laboratory, imaging, and pathological data of included patients were collected from our hospital medical record system. The histological grade was defined by the Ki-67 index and mitotic rate according to the 2019 World Health Organization (WHO) classification of endocrine organ tumors. The TNM stages were defined according to the 8th version of classification system by the AJCC. Functional tumors are defined as having particular symptoms and signs associated with hormone excess, such as insulinoma (hyperinsulinemia) and glucagonoma (hyperglycemia), etc. One lesion was defined as single, and the tumor number ≥2 was defined as multiple.

### 2.2. Follow-Up

The follow-up was conducted by telephone or outpatient visit with a deadline of 30 June 2022. The treatment efficacy was evaluated based on their imaging examination of computed tomography or magnetic resonance imaging. Progression-free survival (PFS) was defined as the duration from the first surgery procedure to the date of tumor progression confirmed radiologically. Overall survival (OS) was defined as the duration from the first surgery procedure to the death or the last follow-up.

### 2.3. Statistical Analysis

All statistical analyses were conducted by SPSS (version 26.0) and Graph-Pad Prism (version 8.0) software. Continuous variables were presented as mean ± standard deviation (SD), and differences between groups were analyzed using *t*-test between two groups and ANOVA analysis among three groups (including the comparison among any two groups). The categorical variables were expressed as frequencies and percentages, and the differences were analyzed by the Chi-square test. Uni-and multivariable Cox proportional hazard regression models were performed to explore the effects of several prognostic factors. The cross-validation was conducted using bootstrap method to demonstrate the predictive ability of FBG-based predictors (bootstrap replicates = 1000). Variables with *p* < 0.05 in univariate analysis were entered into the multivariate analysis. The time-dependent receiver operating characteristic (ROC) curves were plotted and the Youden indices were calculated to determine optimal cutoff value. The Kaplan–Meier method was used to estimate the survival curve which was compared by the log-rank test. A two-tailed *p* < 0.05 was considered to be statistically significant.

## 3. Results

### 3.1. Baseline Characteristics

**Figure 1:**
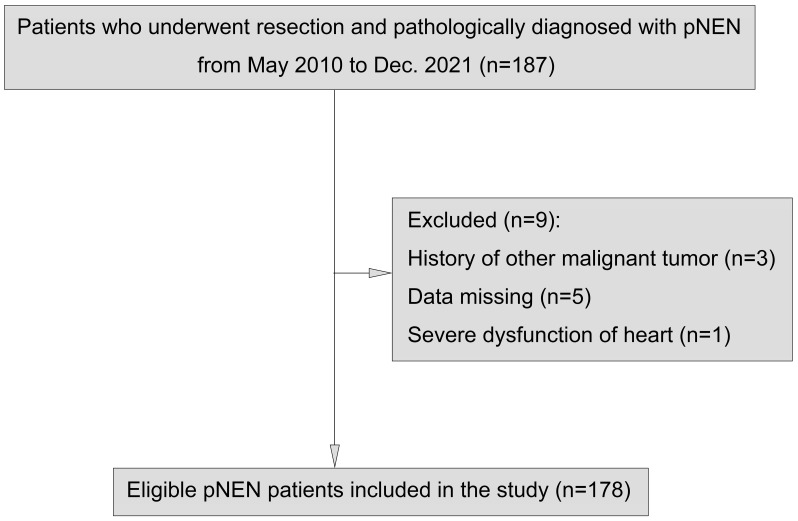
Flow diagram shows patients selection. pNEN, pancreatic neuroendocrine neoplasm.

A total of 187 pNEN patients underwent surgical resection were reviewed, and there were 178 patients eligible for further analyses (Figure 1). The whole cohort comprised 83 (46.6%) males and 95 (53.4%) females, with a mean age of 48.1 ± 13.5 years. The symptoms of hypoglycemia (palpitation, excessive perspiration, tremor, and starvation, etc., n = 58), abdominal bloating (n = 6), and diarrhea (n = 2), etc., were observed in patients with a functional tumor. There were 98 (55.1%), 68 (38.2%), and 12 (6.7%) patients at the histological grade of G1, G2, and G3, respectively, and 134 (75.3%), 17 (9.6%), 6 (3.4%), and 21 (11.8%) patients at the AJCC stages of I, II, III, and IV, respectively. The baseline characteristics of the included patients were summarized in Table 1.

### 3.2. Cutoff Values of Fasting Blood Glucose-Based Novel Indexes

**Figure 2:**
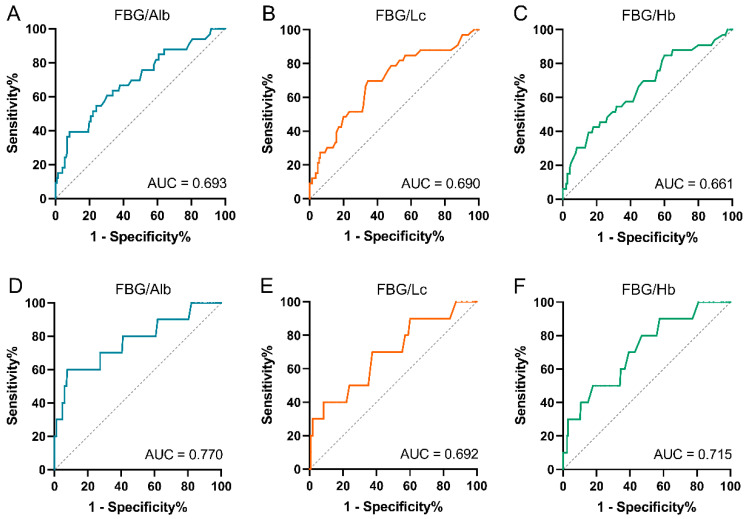
ROC curve analyses of FAR, FLR, and FHR according to PFS and OS. (**A**–**C**) According to PFS, the AUC of FAR, FLR, and FHR was 0.693, 0.690, and 0.661, respectively. (**D**–**F**) According to OS, the AUC of FAR, FLR, and FHR was 0.770, 0.692, and 0.715, respectively. ROC, receiver operating characteristic; AUC, area under the curve; FBG, fasting blood glucose; FAR, FBG-to-albumin ratio; FLR, FBG-to-lymphocytes ratio; FHR, FBG-to-hemoglobin ratio; PFS, progression-free survival; OS, overall survival.

ROC curves were generated to determine the value of FAR, FLR, and FHR for prediction. As for PFS (Figure 2A,C), the area under the curve (AUC) was 0.693 for FAR, 0.690 for FLR, and 0.661 for FHR, respectively. The optimal cutoff value was 0.17 for FAR, 2.85 for FLR, and 0.028 for FHR, respectively. To further assess the predictive efficacy of these cutoff values for OS, ROC curves were also established (Figure 2D,F). The results showed that the AUC was 0.770, 0.692, and 0.715 for FAR, FLR, and FHR, respectively. Additionally, the APAR was also incorporated. To confirm the reliability of these results, the cross-validation was conducted by bootstrap. The bootstrap performances (Supplementary Materials Figure S1) were echoed with the above results. The optimal cutoff value of APAR was 2.2, the AUC was 0.730 for PFS and 0.725 for OS, respectively.

### 3.3. Prediction Roles of Fasting Blood Glucose-Based Indexes for PFS

**Figure 3:**
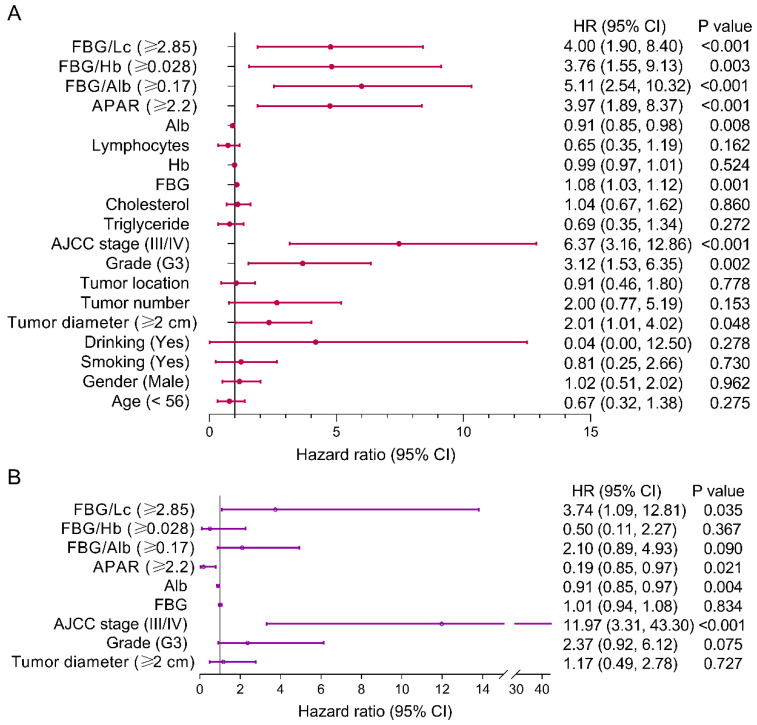
Forest plot showing predictors of PFS in pNEN patients. (**A**) Univariate analysis presented that the FAR, FLR, FHR, and APAR et al. were predictors of PFS in pNEN patients. (**B**) Multivariate analysis showed that the FLR was independent of PFS in pNEN patients. PNEN, pancreatic neuroendocrine neoplasm; FBG, fasting blood glucose; FAR, FBG-to-albumin ratio; FLR, FBG-to-lymphocytes ratio; FHR, FBG-to-hemoglobin ratio; PFS, progression-free survival; APAR, alkaline phosphatase-to-albumin ratio; Alb, albumin; Hb, hemoglobin.

Subgroup analyses were conducted to evaluate the value of FAR, FLR, and FHR in predicting PFS among the above resected groups. As shown in Figure 3, the PFS (median PFS was not applicable) in the low-FAR group (<0.17) was significantly longer than the higher value group (median, 60.0 ± 19.1 months) (*p* < 0.001). The low-FLR group (<2.85) was associated with significantly better PFS compared with the higher value group (*p* < 0.001). Additionally, the PFS in the low-FHR group (<0.028) was also significantly prolonged compared with the high-FHR group (*p* = 0.002).

### 3.4. Risk Factors of PFS for pNEN Patients

**Figure 4:**
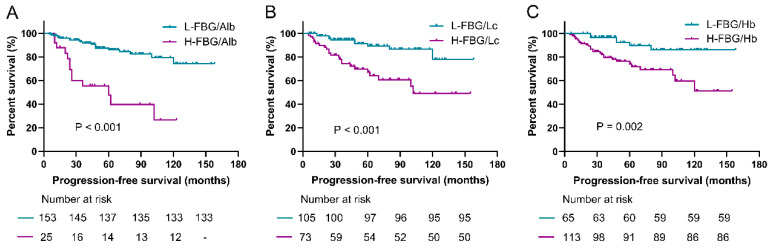
Kaplan–Meier curves of PFS in the high- and low-value groups according to the cutoff values of FAR, FLR, and FHR. (**A**) The median PFS in the low-FAR group was significantly longer compared with the high FAR group (*p* < 0.001). (**B**) The median PFS in the low-FLR group was significantly longer compared with the high FLR group (*p* < 0.001). (**C**) The median PFS in the low-FHR group was significantly longer compared with the high FHR group (*p* = 0.002). FBG, fasting blood glucose; FAR, FBG-to-albumin ratio; FLR, FBG-to-lymphocytes ratio; FHR, FBG-to-hemoglobin ratio; PFS, progression-free survival.

Univariate and multivariate analyses were performed to determine the risk factors of PFS for pNEN patients (Figure 4). The results of univariate analysis showed that the FAR (*p* < 0.001), FLR (*p* < 0.001), FHR (*p* = 0.003), APAR (*p* < 0.001), albumin (*p* = 0.008), FBG, AJCC stage (*p* < 0.001), histological grade (*p* = 0.002), and tumor diameter (*p* = 0.048) were significantly associated with PFS. The results of multivariate analysis demonstrated that the FLR (hazard ratio (HR), 3.74; 95% confidence interval (CI), 1.09, 12.81; *p* = 0.035), APAR (HR, 0.19; 95% CI: 0.85, 0.97; *p* = 0.021), albumin (HR, 0.91; 95% CI: 0.85, 0.97; *p* = 0.004), and AJCC stage (HR, 11.97; 95% CI: 3.31, 43.30; *p* < 0.001) were independent predictors of PFS for pNEN patients.

### 3.5. Prediction Roles of Fasting Blood Glucose-Based Indexes for OS

**Figure 5:**
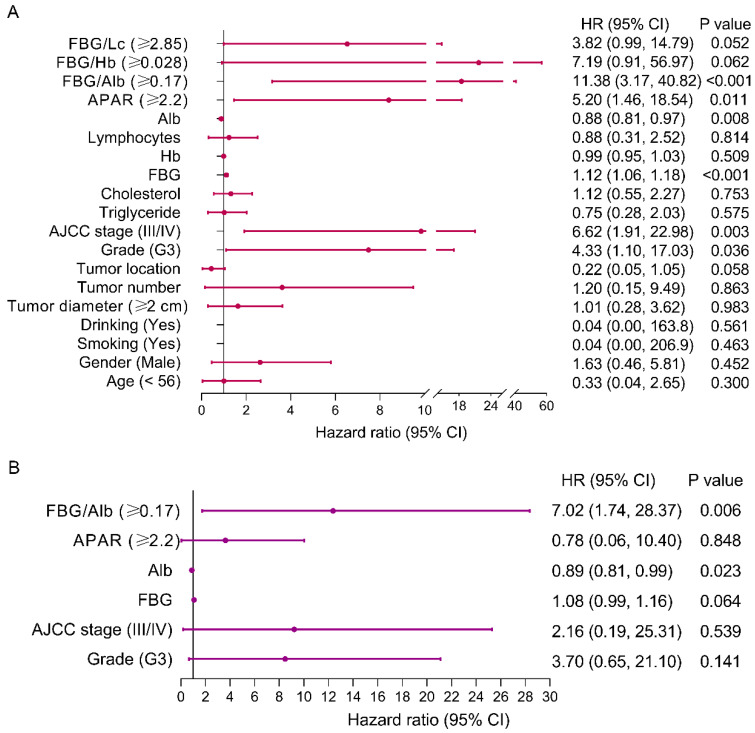
Forest plot showing predictors of OS in pNEN patients. (**A**) Univariate analysis presented that the FAR, APAR, and albumin et al. were predictors of OS in pNEN patients. (**B**) Multivariate analysis showed that the FAR was an independent predictor of OS in pNEN patients. pNEN, pancreatic neuroendocrine neoplasm; FBG, fasting blood glucose; FAR, FBG-to-albumin ratio; OS, overall survival; APAR, alkaline phosphatase-to-albumin ratio; Alb, albumin; Hb, hemoglobin.

As shown in Figure 5, the low-FAR was related to significantly better OS than the higher value group (*p* < 0.001). The OS was significantly prolonged in the low-FLR group compared with the high-FLR group (*p* = 0.037). Additionally, the low-FHR group also showed significantly longer OS than the higher value group (*p* = 0.024).

### 3.6. Risk Factors of OS for pNEN Patients

**Figure 6:**
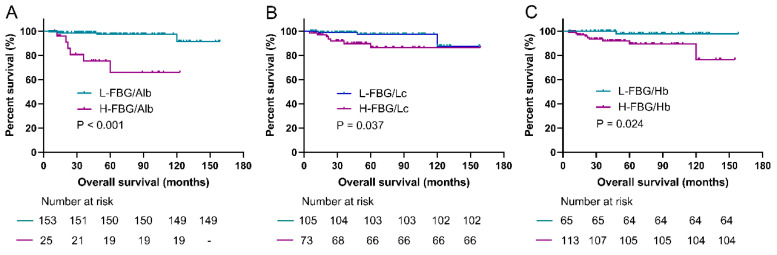
Kaplan–Meier curves of OS in the high- and low-value groups according to the cutoff values of FAR, FLR, and FHR. (**A**) The median OS in the low-FAR group was significantly longer compared with the high FAR group (*p* < 0.001). (**B**) The median OS in the low-FLR group was significantly longer compared with the high FLR group (*p* = 0.037). (**C**) The median PFS in the low-FHR group was significantly longer compared with the high-FHR group (*p* = 0.024). FBG, fasting blood glucose; FAR, FBG-to-albumin ratio; FLR, FBG-to-lymphocytes ratio; FHR, FBG-to-hemoglobin ratio; OS, overall survival.

The results of univariate analyses (Figure 6) presented that the FAR (*p* < 0.001), APAR (*p* = 0.011), albumin (*p* = 0.008), FBG (*p* < 0.001), AJCC stage (*p* = 0.003), and histological grade (*p* = 0.036) were independent predictors of OS for pNEN patients. Further multivariate analyses showed that the FAR (HR, 7.02; 95% CI: 1.74, 28.37; *p* = 0.006) and albumin (HR, 0.89; 95% CI: 0.81, 0.99; *p* = 0.023) were independent predictors of OS.

### 3.7. Baseline Characteristics and Risk Factors of PFS for Patients with pNEN and Metastasis

A total of 30 pNEN patients were concurrent with metastasis, and their demographic characteristics were summarized in Table 2.

The results of univariate analysis of PFS (Table 3) showed that the FAR was the single independent predictor of patients with pNEN and metastasis (HR, 3.34; 95% CI: 1.11, 10.06; *p* = 0.032), and the FBG was the predictor of OS (Table 4) for these patients (HR, 1.15; 95% CI: 1.03, 1.28; *p* = 0.012).

### 3.8. Prediction Role of FAR for Patients with pNEN and Metastasis

**Figure 7:**
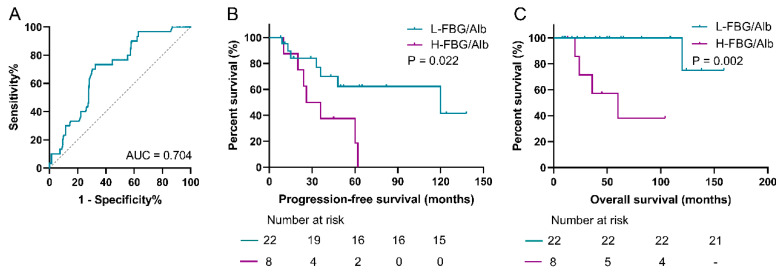
Prediction value of FAR in patients with pNEN and synchronous metastasis. (**A**) ROC curve analysis of FAR showed the AUC was 0.704. (**B**) Kaplan–Meier curves of PFS presented that the low-FAR group was significantly prolonged compared with the high-value cohort (*p* = 0.022). (**C**) Kaplan–Meier curves of OS presented that the low-FAR group was significantly prolonged compared with the high-value cohort (*p* = 0.002). PNEN, pancreatic neuroendocrine neoplasm; FBG, fasting blood glucose; FAR, FBG-to-albumin ratio; ROC, receiver operating characteristic; AUC, area under the curve; PFS, progression-free survival; OS, overall survival.

As for distinguishing the synchronous metastasis in pNEN patients, the ROC curve showed that the AUC was 0.704 (Figure 7). The low-FAR group was correlated with significantly better PFS compared with the higher-value group (*p* = 0.022). Moreover, the lower-value cohort (median, not applicable) also presented significantly prolonged OS when compared with the high-FAR group (median, 60.0 ± 25.1 months) (*p* = 0.002).

## 4. Discussion

The highly heterogeneous prognosis of pNEN patients indicates that risk assessment and the development of new reliable indicators are particularly required [3,10]. Blood markers are regularly measured and easily accessible, which has attracted growing interest and they are widely used in predicting the prognoses of various malignancies. The findings in previous studies are meaningful but still unsatisfactory [9,10]. Therefore, a new prognostic predictor is required to efficiently assess the patient prognosis. Our study provides three novel FBG-based indexes, which firstly, connected FBG with albumin, lymphocytes, and hemoglobin, in effectively predicting the prognosis of pNEN patients, and also identified their metastases prior to treatment. As far as we know, these three indicators have not been previously reported.

Although earlier studies suggested other reliable indexes, such as APAR and neutrophil-to-lymphocyte ratio, there were limitations when they were used as independent factors [5,10]. For instance, in a previous study, APAR was shown to be associated with recurrence-free survival of pNEN patients in the nonmetastatic cohort, but failed to play the prediction role in the multivariate analysis [5]. Moreover, no statistical correlation was found between APAR and OS [5]. In contrast, our study found that the cutoff values of FAR, FLR, and FHR not only significantly distinguished the PFS and OS of the total pNEN patients, the univariate and multivariate analyses also revealed that the FAR was an independent predictor of OS for these patients. The FAR was also an independent factor for detecting patients with pNEN and metastasis according to the multivariate analysis. Additionally, a lower FAR was associated with better PFS and OS in these patients. Therefore, our study demonstrated the effectiveness of pretreatment FAR in detecting metastasis prior to therapy and predicting their prognosis after treatment. Moreover, the FLR was an independent predictor of PFS for the total patients. Although the FHR is effective for predicting PFS and OS in pNEN patients, it was not an independent indicator according to the multivariate analysis. Taken together, our results suggested that the FBG-based indices could be the promising indicators for prognosis and identifying the synchronous metastases, especially combining the FAR with FLR.

Previous research reported that the random blood sugar level was connected with a linearly increased risk of all cancers, and type 2 diabetes mellitus (T2DM) also raised the risk of cancer mortality by 26% [11,12]. In contrast, fasting settings can lead to differential stress sensitization, which makes distinct tumors susceptible to chemotherapy and other therapies while also killing cancer cells as effectively as chemotherapy [13]. Thus, FBG does affect the development of malignancies, and a higher FBG level promotes the progression of cancer. However, the mechanism between FBG and cancer progression remains clarified. The high level of FBG could disturb the homeostatic balance of some endogenous mediators, such as insulin and insulin-like growth factors, which is pro-tumorgenic [14]. Additionally, during the high FBG state, the phosphoinositide-3-kinase (PI-3K), hyperglycemia-associated transcriptional, post-transcriptional factors, and AMP-activated protein kinase probably also contribute to the advancement of cancer [8]. For instance, because the PI-3K singling pathway is crucial for the energy consumption of tumor cells, inhibiting PI-3K could effectively hinder the tumor growth [15]. In hyperglycemia, the integrity of the cellular DNA can be drastically jeopardized, the expression of messenger RNA, long non-coding RNA, and microRNA can be modified, and the post-translational modifications like acetylation can also be altered [16,17,18]. In the present study, the groups of higher FAR, FLR, and FHR all presented worse outcomes as compared with lower cohorts, which echoed with the findings mentioned above.

The nutrition status of a patient plays an important role in cancer development. A prior study demonstrated that the low albumin correlated with a higher risk of negative outcomes [9]. As we all know, cancer cells have a high demand for amino acids to support their proliferation, like serine and glutamine. Serine is necessary for cancer cells to synthesize nucleotide, and glutamine provides the main source of nitrogen to sustain the biosynthesis of new molecules [19,20]. The decomposition products of albumin could provide these amino acids. The progressive cancer cells with chronic wasting biology could consume an amount of albumin, and a lower level of albumin implies a more advanced stage of disease, which was linked to a worse prognosis [21,22]. In addition to the impact on the metabolism, hypoalbuminemia also affects the immunity, such as inducing the cytokines of tumor necrosis factor-a, interleukin-6, and interleukin-1, which promotes the cancer progression [23]. Taken together, hyperglycemia or low albumin means a higher FAR, which is theoretically correlated with poor outcomes. This notion was supported by the findings of our study, which indicated that the low-FAR group had considerably longer PFS and OS.

Hemoglobin also implies the nutritional status to some extent, and recent studies found that hemoglobin is associated with cancer immunity, which attracts growing attention [24,25]. On the one hand, malignancies during progression suppress the synthesis of hemoglobin, and anemia is very common in patients with cancer [22,26]. However, the relationship between hemoglobin and the cancer patients’ outcome has been rarely investigated. The decrease in hemoglobin concentration induces the hypoxic condition, which promotes the invasive phenotype of cancer cells, stimulates neovascularization in tumor tissue via vascular endothelial growth factor signaling pathway, inhibits tumor immune microenvironment, and accelerates cancer progression [26,27,28]. The lower hemoglobin concentration has been reported to be associated with a poor prognosis in certain cancers [29,30]. However, the single parameter of hemoglobin may not be stable enough for prediction [23]. Thereby, a higher hemoglobin concentration, consistent with a lower FBG level, theoretically suggests better outcomes. Our study also demonstrated this hypothesis in which a lower FHR was associated with better outcomes in pNEN patients.

Lymphocytes play a central role in human immunity, which is vital for immunological surveillance and specific anti-tumor immune responses. Higher numbers of lymphocytes may have more reservations and stronger antitumor effects. Research in recent decades has demonstrated that tumor-infiltrating lymphocytes, recruited from circulating blood, are critical for tumor growth. Katz et al. recently demonstrated that a reduced level of tumor-infiltrating lymphocytes is significantly associated with worse PFS in patients with neuroendocrine tumors after resection [31]. Previous study demonstrated that the lymphocyte-based indexes, such as the neutrophil-to-lymphocyte ratio and lymphocyte-to-monocyte ratio, could be effective predictors for various malignancies after treatment [10]. The result of the present study demonstrated that the novel indicator of FHR was significantly correlated with the prognosis of pNEN patients after resection, that is, a lower FHR with better PFS and OS.

Interestingly, our results demonstrated the value of FAR for distinguishing synchronous metastasis in pNEN patients. Metastasis seriously affects the prognosis of pNEN patients [32], and early detection of metastasis could provide important messages for doctors’ decision-making in the following practices, which would help improve the treatment outcomes of these patients. However, the mechanism between a lower FAR and better prognosis requires further investigation.

There is a limitation in the study to be mentioned. This is a single-center retrospective study and selection bias could not be avoided, although the patient number included in this study is large in the pNEN cohort and is followed up in a long-term period.

## 5. Conclusions

Our study provides three novel FBG-based indices, including FAR, FLR, and FHR, which are effective in predicting the prognosis of pNEN patients, as well as detecting their metastases prior to treatment. These markers are inexpensive and easily available in clinical practice, which supports surgeons in making optimal therapy and surveillance strategies for individuals. Moreover, these novel markers have the potential of predicting the outcomes of various other malignancies.

## Figures and Tables

**Table 1:** Baseline characteristics of patients with pNEN.

Characteristics	All (N = 178)	Recover (N = 145)	Progress (N = 33)	*p*-Value
Onset age (years)	48.1 ± 13.5	47.6 ± 13.7	50.6 ± 12.3	0.247
Operative age (years)	50.6 ± 13.0	49.8 ± 13.4	54.0 ± 10.6	0.095
Gender				0.312
Male	83 (46.6%)	65 (44.8%)	18 (54.5%)	
Female	95 (53.4%)	80 (55.2%)	15 (45.5%)	
BMI (kg/m^2^)	23.9 ± 3.7	24.3 ± 3.6	22.2 ± 3.6	**0.003**
Region				0.185
Urban	94 (52.8%)	80 (55.2%)	14 (42.4%)	
Rural	84 (47.2)	65 (44.8%)	19 (57.6%)	
Smoking	21 (11.8%)	18 (12.4%)	3 (9.1%)	0.593
Drinking	12 (6.7%)	12 (8.1%)	1 (3.0%)	0.296
Hypertension	21 (11.8%)	14 (9.7%)	7 (21.2%)	0.063
Diabetes	16 (9.0%)	11(7.6%)	5 (15.2%)	0.170
Tumor				
Diameter (cm)	2.4 ± 1.6	2.2 ± 1.3	3.1 ± 2.3	**0.002**
Number				0.670
Single	164 (92.1%)	133 (91.7%)	31 (93.9%)	
Multiple	14 (7.9%)	12 (8.3%)	2 (6.1%)	
Location				0.791
Head-neck	88 (50.6%)	71 (49.0%)	17 (51.5%)	
Body-tail	90 (49.4%)	74 (51.0%)	16 (48.5%)	
Type				**0.031**
Function	84 (47.2%)	74 (51.0%)	10 (30.3%)	
Nonfunction	94 (52.8%)	71 (49.0%)	23 (69.7%)	
Metastasis				**<0.001**
Yes	30 (16.9%)	0 (0%)	3 (9.1%)	
No	148 (83.1%)	145 (100.0%)	30 (90.9%)	
Ki-67 index	4.4 ± 6.5	3.1 ± 3.6	10.2 ± 11.6	**<0.001**
Histological grade				**<0.001**
G1	98 (55.1%)	89 (61.45)	9 (27.3%)	
G2	68 (38.2%)	53 (36.6%)	15 (45.5%)	
G3	12 (6.7%)	5 (2.1%)	9 (27.3%)	
AJCC stage				**<0.001**
I	134 (75.3%)	132 (91.0%)	2 (6.1%)	
II	17 (9.6%)	13 (9.0%)	4 (12.1%)	
III	6 (3.4)	0 (0%)	6 (18.2%)	
IV	21 (11.8%)	0 (0%)	21 (63.6%)	
FBG (mmol/L)	4.7 ± 3.8	4.1 ± 2.3	7.3 ± 6.9	**<0.001**
Albumin (g/L)	39.2 ± 4.4	39.2 ± 4.5	39.4 ± 3.6	0.803
ALP (U/L)	85.7 ± 56.1	78.6 ± 42.3	117.1 ± 90.0	**<0.001**
Lymphocytes	1.7 ± 0.6	1.7 ± 0.6	1.6 ± 0.7	0.126
Hb	128.2 ± 16.5	128.8 ± 17.0	125.7 ± 13.8	0.335
Cholesterol (mmol/L)	4.4 ± 1.0	4.4 ± 1.1	4.4 ± 0.9	0.964
Triglycerides (mmol/L)	1.4 ± 1.2	1.5 ± 1.2	1.2 ± 0.7	0.315

The bold font represent *p* < 0.05; pNEN, pancreatic neuroendocrine neoplasm; BMI, body mass index; AJCC, American Joint Committee on Cancer; FBG, fasting blood glucose; ALP, alkaline phosphatase; Hb, hemoglobin.

**Table 2:** Baseline characteristics of patients with pNEN and metastasis.

Characteristics	All (N = 30)
Onset age (years)	49.8 ± 11.6
Operative age (years)	52.7 ± 9.9
Gender	
Male	17 (43.3%)
Female	13 (56.7%)
BMI (kg/m^2^)	22.0 ± 3.5
Region	
Urban	14 (46.7%)
Rural	16 (53.3%)
Smoking	2 (6.7%)
Drinking	1 (3.3%)
Hypertension	3 (10.0%)
Diabetes	3 (10.0%)
Tumor	
Diameter (cm)	3.1 ± 2.4
Number	
Single	28 (93.3%)
Multiple	2 (6.7%)
Location	
Head-neck	14 (46.7%)
Body-tail	16 (53.3%)
Type	
Function	9 (30.0%)
Nonfunction	21 (70.0%)
Ki-67 index	10.1 ± 11.9
Histological grade	
G1	8 (26.7%)
G2	14 (46.7%)
G3	8 (26.7%)
AJCC stage	
I	0
II	0
III	6 (20.0%)
IV	21 (70%)
FBG (mmol/L)	6.6 ± 5.4
Albumin (g/L)	39.4 ± 3.8
ALP (U/L)	120.0 ± 93.9
Lymphocytes	1.6 ± 0.7
Hb	126.1 ± 13.7
Cholesterol (mmol/L)	4.4 ± 0.9
Triglycerides (mmol/L)	1.2 ± 0.7

PNEN, pancreatic neuroendocrine neoplasm; BMI, body mass index; AJCC, American Joint Committee on Cancer; FBG, fasting blood glucose; ALP, Alkaline phosphatase; Hb, hemoglobin.

**Table 3:** Univariate and multivariate analysis of PFS for patients with pNEN and metastasis.

Characteristics	Univariate Analysis		Multivariate Analysis	
	HR (95% CI)	*p*-Value	HR (95% CI)	*p*-Value
Age	0.88 (0.24, 3.20)	0.845		
<56				
≥56 *				
Gender	0.50 (0.17, 1.44)	0.199		
Male				
Female *				
Smoking	0.04 (0.00, 178.2)	0.448		
Yes				
No *				
Drinking	0.04 (0.00, 178.2)	0.448		
Yes				
No *				
Tumor				
Diameter	0.48 (0.19, 1.18)	0.109		
<2 cm				
≥2 cm *				
Number	1.56 (0.20, 12.28)	0.671		
Location	1.70 (0.58, 4.97)	0.337		
Head-Neck				
Body-Tail *				
Grade	1.30 (0.36, 4.70)	0.685		
G1/2				
G3 *				
AJCC stage	32.51 (0.12, 9220)	0.227		
I/II				
III/IV *				
Triglyceride				
Cholesterol				
FBG	1.06 (0.98, 1.14)	0.152		
Hb	0.99 (0.96, 1.03)	0.712		
Lymphocytes	0.82 (0.37, 1.79)	0.611		
Alb	0.99 (0.86, 1.14)	0.880		
APAR	1.53 (0.48, 4.88)	0.476		
<2.2				
≥2.2 *				
FBG/Alb	3.34 (1.11, 10.06)	**0.032**	3.34 (1.11, 10.06)	**0.032**
<0.17				
≥0.17 *				
FBG/Hb	2.13 (0.27, 16.59)	0.470		
<0.028				
≥0.028 *				
FBG/Lc	2.02 (0.62, 6.55)	0.243		
<2.85				
≥2.85 *				

*, reference index; The bold font represent *p* < 0.05; PFS, progression-free survival; HR, hazard ratio; CI, confidence interval; AJCC, American Joint Committee on Cancer; FBG, fasting blood glucose; Hb, hemoglobin; Alb, albumin; APAR, alkaline phosphatase-to-albumin ratio; Lc, lymphocytes.

**Table 4:** Univariate and multivariate analysis of OS for patients with pNEN and metastasis.

Characteristics	Univariate Analysis		Multivariate Analysis	
	HR (95% CI)	*p* Value	HR (95% CI)	*p* Value
Age	2.30 (0.38, 13.86)	0.365		
<56				
≥56 *				
Gender	0.31 (0.05, 1.88)	0.204		
Male				
Female *				
Smoking	0.04 (0.00, 20,227)	0.626		
Yes				
No *				
Drinking	0.04 (0.00, 20,227)	0.626		
Yes				
No *				
Tumor				
Diameter	0.84 (0.21, 3.33)	0.799		
<2 cm				
≥2 cm *				
Number	0.05 (0.00, 355,142)	0.738		
Location	6.21 (0.67, 57.56)	0.108		
Head-Neck				
Body-Tail *				
Grade	1.58 (0.18, 14.25)	0.685		
G1/2				
G3 *				
AJCC stage	29.18 (0.00, 41,576)	0.489		
I/II				
III/IV *				
Triglyceride				
Cholesterol				
FBG	1.15 (1.03, 1.28)	**0.012**		
Hb	1.03 (0.96, 1.10)	0.448		
Lymphocytes	0.88 (0.25, 3.03)	0.837		
Alb	1.10 (0.88, 1.38)	0.392		
APAR	2.38 (0.26, 21.60)	0.441		
<2.2				
≥2.2 *				
FBG/Alb	250.7 (0.01, 59,184)	0.282		
<0.17				
≥0.17 *				
FBG/Hb	27 (0.00, 1,398,079)	0.552		
<0.028				
≥0.028 *				
FBG/Lc	3.32 (0.37, 30.07)	0.285		
<2.85				
≥2.85 *				

*, reference index; The bold font represent *p* < 0.05; OS, overall survival; HR, hazard ratio; CI, confidence interval; AJCC, American Joint Committee on Cancer; FBG, fasting blood glucose; Hb, hemoglobin; Alb, albumin; APAR, alkaline phosphatase-to-albumin ratio; Lc, lymphocytes.

## Data Availability

The data can be obtained from the corresponding author upon requirement.

## Supplementary Materials

The following supporting information can be downloaded at: https://www.mdpi.com/article/10.3390/jpm14070760/s1, Figure S1: Bootstrapped ROC performance of FAR, FLR, and FHR according to PFS and OS.

## Abbreviations

PNENs	pancreatic neuroendocrine neoplasms
AJCC	American Joint Committee on Cancer
ALP	alkaline phosphatase
Alb	albumin
APAR	alkaline phosphatase to albumin ratio
OS	overall survival
FBG	fasting blood-glucose
CRP	C-reactive protein
FAR	FBG-to-albumin ratio
FLR	FBG-to-lymphocytes ratio
FHR	FBG-to-hemoglobin ratio
WHO	World Health Organization
PFS	Progression-free survival
SD	mean ± standard deviation
ROC	receiver operating characteristic
