# Supplementary material for: Fasting Blood Glucose-Based Novel Predictors in Detecting Metastases and Predicting Prognosis for Patients with PNENs
Source: J Pers Med. 2024 Jul 17;14(7):760. doi: 10.3390/jpm14070760 (PMC11277919; doi:10.3390/jpm14070760)
Supplement: Supplementary file 1 [file jpm-14-00760-s001.zip › jpm-3044848-supplementary.pdf]

Supplementary Material

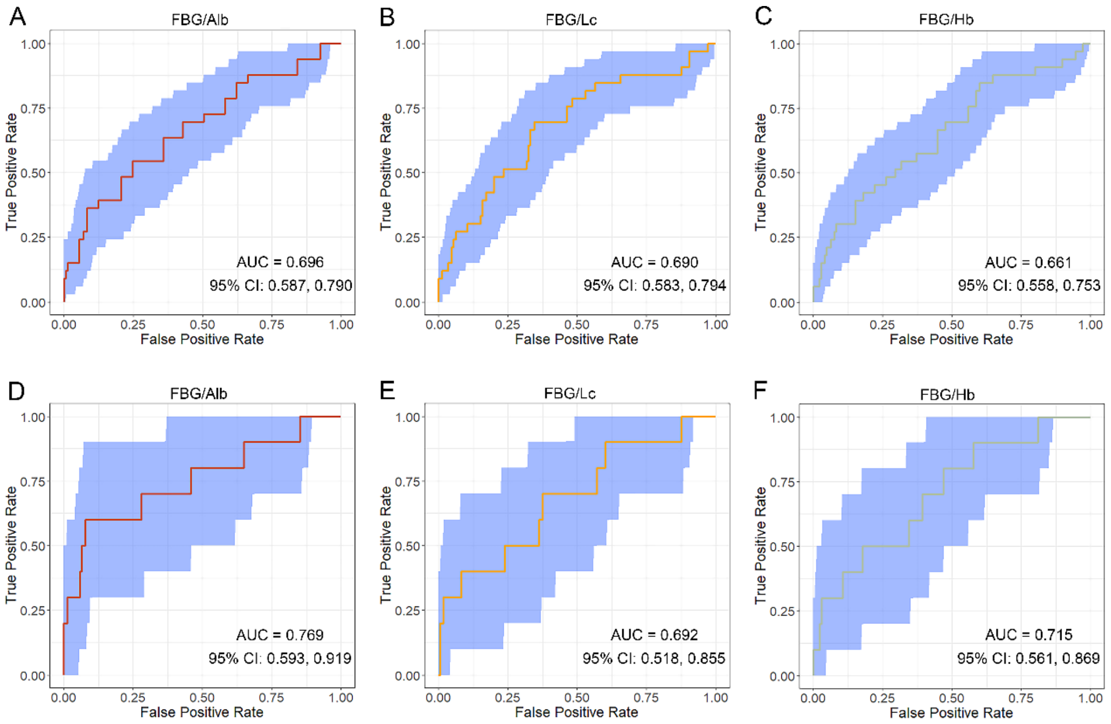

Figure S1. Bootstrapped ROC performance of FAR, FLR, and FHR according to PFS and OS. (A-C) According to PFS, the AUC of FAR, FLR, and FHR was 0.696, 0.690, and 0.661, respectively. (D-F) According to OS, the AUC of FAR, FLR, and FHR was 0.769, 0.692, and 0.715, respectively. ROC, receiver operating characteristic; AUC, area under the curve; FBG, fasting blood glucose; FAR, FBG-to-albumin ratio; FLR, FBG-to-lymphocytes ratio; FHR, FBG-to-hemoglobin ratio; PFS, progression-free survival; OS, overall survival.
